# Associations between Lake Urmia disaster and the prevalence of thyroid nodules and metabolic syndrome: The AZAR cohort survey

**DOI:** 10.34172/hpp.2022.40

**Published:** 2022-12-10

**Authors:** Jalil Houshyar, Alireza Ostadrahimi, Samira Pourmoradian, Elnaz Faramarzi, Helda Tutunchi, Majid Mobasseri

**Affiliations:** ^1^Endocrine Research Center, Tabriz University of Medical Sciences, Tabriz, Iran; ^2^Nutrition Research Center, Tabriz University of Medical Sciences, Tabriz, Iran; ^3^Department of Community Nutrition, Faculty of Nutrition and Food Science, Tabriz University of Medical Sciences, Tabriz, Iran; ^4^Liver and Gastrointestinal Diseases Research Center, Tabriz University of Medical Sciences, Tabriz, Iran

**Keywords:** Metabolic syndrome, Lake Urmia disaster, Thyroid nodules

## Abstract

**Background:** In this study, we investigated the associations Lake Urmia’s drought to the prevalence of thyroid nodules (TNs) and metabolic syndrome (MetS) among local inhabitants of the lake.

**Methods:** In this cross-sectional study which was started in 2014, we collected data on 992 adults who participated in the Azar cohort study, in Shabestar county, Iran. The sociodemographic status, smoking, and medical history of the subjects living in the areas adjacent to (n = 163) and far from (n = 829) Lake Urmia were collected through questionnaires. After obtaining written consent, anthropometric factors and blood pressure (BP) were measured. The lipid profile and fasting blood glucose (FBG) of the respondents were measured using colorimetric methods, and all underwent thyroid examination and sonography. Furthermore, the size and characteristics of nodules were determined with a fine-needle aspiration biopsy (FNAB) method.

**Results:** We did not find any significant difference in the prevalence of TNs between the two groups (*P*=0.44), whereas the prevalence of MetS were significantly higher among the subjects from the regions that were far from the Lake (*P*=0.04). After adjustment for confounding factors (age and gender) in both groups, low risk of TNs (OR=1.20, 95% CI:0.89-1.62) and high risk of TNs (OR=1.19, 95% CI:0.65-2.19) were not significantly associated to MetS (*P*>0.05).

**Conclusion:** In this study, Lake Urmia’s drought was identified to be with no contribution to the prevalence of TNs and MetS. Therefore, long term perspective studies are suggested to reach precise results.

## Introduction

 Thyroid nodule (TN) is a common adult disease which increases as the population ages. While most nodules are benign, 8–15% prove to be cancerous. The thyroid cancer is the fourth leading type of cancer worldwide, and the age-standardized incidence rate of the cancer in Iran was 1.57 in 1990, which increased by 131%, until 2019. The age-standardized prevalence rate of thyroid cancer was 30.19 in 2019,which increased by 164% from 11.44 in 1990.^1^ This global increase is due to numerous factors, including increased detection of primary tumors, malignant TNs, high prevalence of modifiable individual risk factors (e.g., obesity), and increased exposure to environmental risk factors, such as climate changes.^2^ A previous systematic review indicated that the drying of Aral saline lake increased the prevalence of several diseases, such as respiratory diseases, asthma, eye diseases, cancers, typhoid, hepatitis, arthritis, impaired endocrine function, neurological and behavioral changes, immune system diseases, mental retardation, and delayed puberty.^3^

 In northwest of Iran, Lake Urmia, the second largest saline lake, is drying due to several reasons, including climate change, excessive groundwater extraction, lack of efficient water management, industrial and domestic overuses, dam constructions and devoting uncontrolled water sources for agricultural use.^4^

 Lake Urmia’s drought seems to be a serious threat to the environment, the health of people, local economy, and food security. The ecosystem of the area around the lake might be completely destroyed in parallel with the lake drought. It is shown that Lake Urmia’s drought created several centers of salt dust, and affected the agriculture, economy and public health of the area.^2^ A recent study conducted by Sadegh Tabrizi et.al showed that Lake Urmia’s drought has had serious effects on hypertension and anemia in the individuals living near to the lake, but it has not had significant effects on the prevalence of hyperlipidemia, overweight/obesity, asthma, angina, infraction, diabetes, and vitamin D insufficiency/deficiency in the area.^5^

 In the past 30 years, TNs have become a common thyroid disorder worldwide, which may increase the possibility of thyroid cancer. Therefore, the risk factors associated with TNs have received significant attention, in recent years. A variety of metabolic diseases are considered to be important risk factors for TNs.^6^ Several reports have demonstrated a positive association between metabolic syndrome (MetS) and the prevalence of TNs. It was hypothesized that compensatory hyperinsulinemia following insulin resistance in previous MetS might be responsible for the rising trend of TNs.^7^ On the other hand, a previous study found that TNs were significantly related to an increased risk of MetS. Insulin resistance can directly activate the proliferation pathway, through insulin or insulin-like growth factor 1, regulating the expression of thyroid genes, and the proliferation and differentiation of thyroid cells. In addition, insulin resistance may also be related to thyroid vascular structure.^8^

 To the best of our knowledge, there is no study that has examined the contribution of Lake Urmia’s drought to the prevalence of TNs and MetS. Therefore, the aim of the present study was to examine the association between Lake Urmia’s drought and the prevalence of TNs and MetS in the inhabitants of the area close to Lake Urmia.

## Material and Methods

###  Participants and procedures 

 In this cross-sectional study, 2000 adult subjects aged 35-70 years were recruited. The Azar Cohort study is a part of the large Iranian prospective epidemiological research study (Persian Cohort),^9^ explained with more details elswhere.^10^

 Multi-stage stratified random sampling was applied to select participants from five villages near/adjunct the lake (adjunct area at least 5 km), and six villages far from the lake areas. Eligible participants were invited through telephone call to take part in the survey.

 The research objectives and protocol were explained to the participants, and all signed written consent forms. Participants with the history of thyroid diseases were excluded. Thyroid examination and thyroid ultrasonography were performed by a trained general practitioner, according to the guideline developed for the cohort.

###  Anthropometric factors, MetS components, and blood sampling

 Using National Institute of Health (NIH) guidelines, anthropometric factors, including body weight, height, and waist circumference were measured, and body mass index (BMI) was calculated using the formula kg/m^2^.^11^ Blood pressure (BP) was measured twice a day with 2-minute intervals in each arm in a sitting position after a 10-minute rest period using a mercury sphygmomanometer (Rudolf Richter; DE-72417; Germany). The averages of these two measurements were used as the daily systolic and diastolic BP measurements.^12^

 Blood samples were collected after a 10–12 hours overnight fast. The fasting blood glucose (FBG), triglyceride (TG), total cholesterol (TC), high-density lipoprotein cholesterol (HDL-C), and low-density lipoprotein cholesterol (LDL-C) were determined by enzymatic methods.

###  Metabolic syndrome definition 

 In this study, the National Cholesterol Education Program Adult Treatment Panel III report criteria (ATP III) was used to select participants with MetS. At least, three of the following criteria were defined as MetS: waist circumference ≥ 102 cm for men and ≥ 88 cm for women, TG ≥ 150 mg/dL or drug treatment for elevated TG as an alternate indicator, and HDL-C values of < 40 mg/dL for men and < 50 mg/dL for women. Hypertension was defined as systolic BP ≥ 130 mm Hg and/or diastolic BP ≥ 85 mm Hg or the use of antihypertensive medications. Elevated FBG was considered to be ≥ 100 mg/dL or the use of glucose-lowering medications.^13^

###  Thyroid nodule evaluation

 Ultrasound is the preferred imaging modality for TNs. Participant underwent thyroid examination and sonography using a device with a 7.5 MHz probe. Furthermore, the size and characteristics of nodules were determined with fine-needle aspiration biopsy (FNAB) method. FNAB is the accepted diagnostic test to determine whether a TN is benign or malignant. Nodules with 1 cm of diameter and larger, and/or nodules with suspicious sonographic appearance warrant cytological analysis to better quantify the risk for malignancy.^14^

###  Statistical analysis

 Descriptive statistics were performed to explore mean and standard deviation (SD) for continuous variables and prevalence (%) for categorical variables, respectively. The normality of the continuous variables was examined using descriptive statistics. The independent sample t-test and chi-square test were used for between-group comparisons. The relationships between prevalence of TNs, MetS and study variables were assessed using binary logistic regression analysis. The odds ratios (ORs) and 95% confidence intervals (CIs) were obtained using logistic regression analyses with/without adjustments for age and gender. A *P* value less than 0.05 was considered as statistically significant. Data were analyzed using IBM SPSS Statistics (version 20; IBM Corp, Armonk, NY).

## Results

 General characteristics of the participants according to distance from Lake Urmia were presented in Table 1. The overall prevalence of TNs was 37% (42.2% female and 30.6% male), and the age range of participants with TNs was from 60 to 69 years. Multi-nodular (more than two nodules) was 46.9% (61.8% female vs. 39.4% male) among the patients with ≥ 60 years of age.

**Table 1:** General characteristics of participants according to distance from Lake Urmia

	**Lake Urmia adjacent** **(n=163)**	**Areas far from the Lake (n=829)**	***P***
	**N (%)**	**N (%)**	
**Gender**			0.38^a^
Male	81 (49.7)	375 (45.2)	
Female	82(50.3)	454 (54.8)	
**Marital status**			0.06^a^
Married	160 (98.2)	785 (94.7)	
No married	3 (1.8)	44 (5.3)	
**Education level**			0.01^a^
illiterate	40(24.5)	144(17.4)	
Primary school	86(52.8)	392(47.3)	
Middle school	34(20.9)	269(32.4)	
University education	3(1.8)	24(2.9)	
**Socioeconomic status**			0.21^a^
Very high	16(9.9)	100(12.1)	
High	32(19.7)	168(20.3)	
Middle	27(16.5)	193(23.3)	
Low	42(25.7)	172(20.6)	
Very low	46(28.2)	196(23.7)	
**Current smoking status**			0.28^a^
Daily	21(12.9)	86(10.4)	
Occasionally	1(0.6)	18 (2.2)	
NO	141(86.5)	725(87.5)	
**Medical History**			
Diabetes	88(10.6)	20(12.3)	0.58^a^
Hypertension	166(20)	42(25.8)	0.11
Thyroid nodule risk			0.44^a^
No risk	103(63.2)	523(63.1)	
low risk	55(33.7)	254(30.6)	
high risk	5(3.06)	52(6.3)	
**Metabolic syndrome**	276(33.5)	68(41.7)	0.04^a^
	**Mean ±SD**	**Mean ±SD**	
Age	52.83±9.26	53.10±9.51	0.71^b^
Weight (kg)	75.45±14.27	77.68±14.31	0.06^b^
BMI (kg/m^2^)	28.83±5.21	29.53±5.00	0.11^b^

^a^ χ2 test; ^b^Independent *t* test.

 As presented in Table 1, there was no significant difference in distance from Lake Urmia by demographic characteristics, except for education level (*P* = 0.01). However, the risk of TNs, diabetes, and hypertension was the same in both studied group**s** (*P* > 0.05). The prevalence of MetS was significantly higher in the regions far from the Lake Urmia in comparison with the regions near to the lake (*P* = 0.04).

 The association between TNs risk (low risk vs. no risk participants) and MetS by distance from Lake Urmia, using binary logistic regression analysis, is shown in Table 2. The reference group in our analysis was “far from the lake group”. In the region near to the Lake Urmia, the risk of TNs was decreased insignificantly (OR: 0.9, 95% CI: 0.71-4.76). The association remains insignificant after adjustment for confounding factors (age and gender) (OR: 0.89, 95% CI: 0.61-1.30). The MetS increased significantly in participants with low risk of TNs, compared to those without the risk of TNs in “the near to the lake region” (OR: 1.53, 95% CI: 1.15-2.03). After adjustment for confounding factors (age and gender), the association was still statistically insignificant (OR:1.20, 95% CI: 0.89-1.62).

**Table 2:** The association between thyroid nodules and metabolic syndrome in low risk versus no risk individual according to distance from Lake Urmia

**Variables **	**Model 1**			**Model 2**		
	**OR**	**95% CI**	***P***	**OR**	**95% CI**	***P***
Far from the Lake Urmia	0.91	0.63-1.30	0.60	0.89	0.61-1.30	0.56
Metabolic syndrome	1.53	1.15-2.03	0.003	1.20	0.89-1.62	0.22

OR, odd ratio; CI, confidence interval. Model 1. Unadjusted model, Model 2. Adjusted for age, gender. *Note:* Results of logistic regression model (the far from the lake consider as reference group).

 The association between TNs risk (high risk vs. no risk participants) and MetS by distance from Lake Urmia, using binary logistic regression analysis, is shown in Table 3.In “the region near to the Lake Urmia”, the risk of TNs was increased insignificantly (OR:1.85, 95% CI: 0.71-4.76) in the unadjusted model. The association was insignificant after adjustment for confounding factors (age and gender) (OR: 1.73, 95% CI: 0.66-4.48). MetS increased insignificantly in the patients with the high risk of TNs in comparison with the participants without the risk of TNs in “near to the lake region” (OR: 1.48,95% CI; 0.83-2.64). Also, the association was statistically insignificant (OR: 1.19, 95% CI: 0.65-2.19) after adjustment for confounding factors (age and gender).

**Table 3:** The association between thyroid nodules and metabolic syndrome in high risk versus no risk individual according to distance from Lake Urmia

**Variables **	**Model 1**			**Model 2**		
	**OR**	**95% CI**	***P***	**OR**	**95% CI**	***P***
Far from the Lake Urmia	1.85	0.71-4.76	0.20	1.73	0.66-4.48	0.25
Metabolic syndrome	1.48	0.83-2.64	0.18	1.19	0.65-2.19	0.55

OR, odd ratio; CI, confidence interval. Model 1. Unadjusted model, Model 2. Adjusted for age, gender. *Note:* Results of logistic regression model (the far from the lake consider as reference group).

## Discussion

 Based on the results of this study, the overall prevalence of TNs was 37% (42.2% female and 30.6% male), and the age range of participants with TNs was from 60 to 69 years. Multi-nodular (more than two nodules) was 46.9% (61.8% female vs. 39.4% male) in the patients with ≥ 60 years of age. These findings were in accordance with the results of Jiang et al, who reported the prevalence of TNs to be 49% (42.7 % males, 52.5% females), among South Korean patients with 60-69 years of age.^15^ However, in the previous studies conducted by Sanei Taheri et al^16^ and Rezaei-Delui et al,^17^ the prevalence of TNs in Iran was reported to be 52.3% and 28.6%, respectively. The statistical differences found in the prevalence of TNs in these studies may be due to the differences in evaluation time, age and gender of target populations, and iodine status (iodine deficiency) of the studied population by their living regions.^2,18^

 In the present study, we found age and gender in association with the prevalence of TNs. The prevalence of TNs in the patients with 70 years of age and older was higher than those in the patients with less than 70 years of age. In the present study and most of previous researches, the prevalence of TNs is reported to be higher in females. The reason for such a higher incidence of TNs in women might be related to the effect of estrogen on thyroid stimulating hormone. The effects of thyroid tissue and thyroid neoplasty on the expression of estrogen receptors in these tissues may indicate the role of estrogen on the growth of TNs.^19,20^ Previous clinical studies confirmed these results, based on the frequency of nodules in pregnant and infertile women.^21,22^

 Several studies have revealed that geographical distance from sea and climate change have serious health outcomes, and may alter the prevalence of several chronic diseases.^23,24^ Our study findings showed that the Lake Urmia’s drought had no contribution to the prevalence of TNs, however, the prevalence of MetS was significantly high in the region far from the lake. In accordance with our results, Sadegh Tabrizi et al evaluated the effects of Lake Urmia’s drought on the health of residents near to the lake, and found significant differences in the mean levels of serum TC, LDL-C, HDL-C, and TG between those from the Lake Urmia’s adjacent areas and their control group.^5^

 In the present study, we did not find any difference in the prevalence of hypertension and BMI by residency in these two areas. Sadegh Tabrizi et al also found no significant difference in the prevalence of type 2 diabetes and obesity between the two groups, however, the prevalence of hypertension and prehypertension was significantly higher in the regions near to the lake.^5^ Such controversial findings between their study and ours may be explained by the difference in population groups in each study. For instance, Sadegh Tabrizi et al, only included the participants from urban areas, but we included participants from both urban and rural areas. Furthermore, they conducted sampling only from three cities in each area, but we selected five to six villages/cities in each group.

 In our study, even after adjustment for confounding factors (age and gender), no association was found between the risk of TNs and MetS in both groups. Previous reports suggest the components of MetS as possible risk factors for TNs. MetS is thought to trigger TNs via the stimulation of thyroid proliferation and angiogenesis induced by hyperinsulinemia, hyperglycemia, and dyslipidemia.^25,26^In another study, the risk of TNs increased by increase in MetS components.^27^ In our study, however, we found no significant association between MetS and TNs, which may be justified by the differences in demographic characteristics and iodine status of the studied populations. Zhang et al in a recent meta-analysis revealed uncertainty on whether this association will be sustained in population with different demographic characteristics. They, also, reported that iodine has a crucial impact on the prevalence of thyroid diseases and MetS. Their findings, eventually, showed no association between TNs and MetS in iodine-deficient populations.^8^

###  Strengths and limitations

 As a strength, the present study is the first research that examined the effects of Lake Urmia’s drought on the prevalence of TNs and MetS. However, the cross-sectional design of the study might be considered as a limitation, and thus we warrant cause-effect relationship between Lake Urmia’s drought and metabolic changes in this population. Longitudinal studies are needed to confirm our results.

## Conclusion

 In conclusion, for the first time, we revealed that Lake Urmia’s drought is not contributed to the prevalence of TNs and MetS among the population living in the lake area. Further studies with long-term perspectives are recommended to reach precise results.

## Acknowledgements

 The authors are deeply indebted to all the patients who participated in this study. This work was financially supported by Endocrine Research Center, Tabriz University of Medical Sciences. We thank the contribution by the investigators and the staff of the Azar cohort study.

## Author Contributions

**Conceptualization:** Alireza Ostadrahimi, Majid Mobasseri.

**Data curation: **Jalil Hoshyar.

**Formal Analysis: **Elnaz Faramarzi.

**Funding acquisition: **Majid Mobasseri.

**Investigation: **Jalil Hoshyar, Elnaz Faramarzi.

**Methodology: **Majid Mobasseri.

**Project administration:** Jalil Hoshyar, Elnaz Faramarzi.

**Resources: **Alireza Ostadrahimi.

**Software:** Samira Pourmoradian, Elnaz Faramarzi.

**Supervision: **Majid Mobasseri.

**Validation: **Elnaz Faramarzi.

**Visualization: **Samira Pourmoradian.

**Writing – original draft: **Samira Pourmoradian, Elnaz Faramarzi.

**Writing – review & editing:** Elnaz Faramarzi, Helda Tutunchi.

## Funding

 Endocrine Research Center of Tabriz University of Medical Sciences, Tabriz, Iran and the Liver and Gastrointestinal Diseases Research Center of Tabriz University of Medical Sciences supported this study (Grant number 700/108).

## Ethical Approval

 The present study was approved by Ethics Committee in Tabriz University of Medical Sciences (Ethics code: IR.TBZMED.REC.1397.778).

## Competing Interests

 The authors declare no conflict of interest.
